# Levels of Evidence Supporting Recommendations in Gastroenterology

**DOI:** 10.14309/ctg.0000000000000797

**Published:** 2024-12-06

**Authors:** Miyabi Saito, Amy Yu, Nneka N. Ufere, Andrew Chan, Bharati Kochar

**Affiliations:** 1 Department of Medicine, Massachusetts General Hospital, Boston, Massachusetts, USA;; 2 Harvard Medical School, Boston, Massachusetts, USA;; 3 Division of Gastroenterology, University of California San Francisco, San Francisco, California, USA;; 4 Division of Gastroenterology, Massachusetts General Hospital, Boston, Massachusetts, USA;; 5 Clinical & Translational Epidemiology Unit, The Mongan Institute, Boston, Massachusetts, USA.

**Keywords:** guidelines, guidance, evidence-based medicine, hepatology

## Abstract

**INTRODUCTION:**

We aimed to analyze gastrointestinal guidelines to assess the quality of evidence and strength of recommendation.

**METHODS:**

We abstracted clinical practice guidelines and guidance statements from 4 American gastroenterology societies (American College of Gastroenterology, American Gastroenterological Association, American Society for Gastrointestinal Endoscopy, and American Association for the Study of Liver Disease) and the US Multi-Society Task Force.

**RESULTS:**

Of the 3,609 statements analyzed, only 13% were supported by high level of evidence. The number of statements published annually is increasing, but the level of evidence supporting recommendations is declining over time.

**DISCUSSION:**

This analysis highlights the need for high quality research in gastroenterology to support the development of stronger evidence-based guideline statements.

## INTRODUCTION

Clinical guidelines are formulated to guide evidence-based medical decision making (1). Formalized processes exist to assess the strength and level of evidence, supporting a recommendation. A leading method used in the United States is the Grading of Recommendations, Assessment, Development, and Evaluation (GRADE) method, which is a standardized system for rating both the quality of evidence and strength of recommendations (2). The levels of quality of evidence range from high, which are those backed by randomized controlled trials (RCT), to very low, which are generally based on expert consensus. Strengths of recommendations are assessed on level of evidence and incorporate factors including the comparator, the perspective, and the health care setting (3). Ratings for strengths of recommendations vary from strong to weak.

A study of 39 guidelines published by the American College of Gastroenterology concluded that >50% of recommendations were based on low quality evidence (4). However, this analysis was based on a single society and included a limited number of guidelines. In this study, we aimed to analyze a broader repertoire of statements published by the leading American organizations that publish guidelines relevant to gastroenterology (GI) practice.

## METHODS

Clinical practice guidelines were identified as guidelines and guidance statements published on the websites of 4 prominent GI societies: American College of Gastroenterology, American Gastroenterological Association, American Society for Gastrointestinal Endoscopy, American Association for the Study of Liver Diseases, and US Multi-Society Task Force, from January 1, 2009, to September 30, 2023. Guidelines were characterized as statements which used objective evidence classification (GRADE or American Heart Association (AHA)/American College of Cardiology (ACC) criteria), whereas guidance statements were those without formal evidence classification. Expert consensus documents, performance measures, and focused updates were not included. COVID-19-related statements were also excluded. Each statement was abstracted and categorized by publication year, society, and primary GI subspeciality. For each guideline and guidance statement in each subspeciality, we documented the level of evidence and strength of recommendation.

We determined the number and proportion of guideline recommendations in each subspeciality and those classified into each of the GRADE categories for levels of evidence (high, moderate, low, very low) and strength of recommendation (strong, conditional). This was similarly conducted for the AHA/ACC methodology-based statements characterized as levels A (highest level) through C (lowest level).

## RESULTS

**Figure 1.**
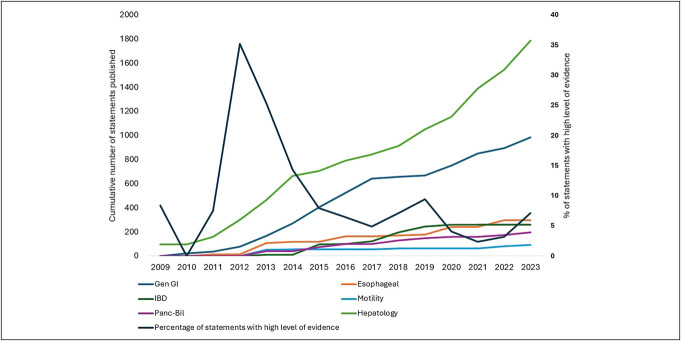
Cumulative number of guideline and guidance statements published by gastrointestinal subspecialty and the percentage of statements each year with high level of evidence. GI, gastroenterology.

From 168 publicly published guidelines (n = 140) and guidance (n = 28) documents, we abstracted 3,609 statements (Table 1). Of these, 9 were updates of previously published statements. The number of published statements sharply increased over time (Figure 1). Hepatology had the greatest number of statements (50%), followed by general GI (27%), esophageal (8%), inflammatory bowel disease (IBD) (7%), pancreaticobiliary (5%), and motility (3%).

**Table 1.** Statements by subspeciality and methodology

Subspeciality	Total # of statements	% guidelines	GRADE	AHA/ACC	% guidance
Gen GI	982	89.9% (883/982)	87.8% (863/883)	0	10.1% (99/982)
IBD	260	100% (260/260)	100% (260/260)	0	0
Motility	91	100% (91/91)	100% (91/91)	0	0
Pancreaticobiliary	195	88.2% (172/195)	100% (172/172)	0	11.8% (23/195)
Hepatology	1,786	56.6% (1,010/1,786)	91.9% (928/1,010)	8.1% (82/1,010)	43.4% (776/1,786)
Esophageal	295	100% (295/295)	100% (295/295)	0	0
Total	3,609	2,711	2,609	82	898

AHA/ACC, American Heart Association/American College of Cardiology; GI, gastroenterology; GRADE, Grading of Recommendations, Assessment, Development, and Evaluation; IBD, inflammatory bowel disease.

**Figure 2.**
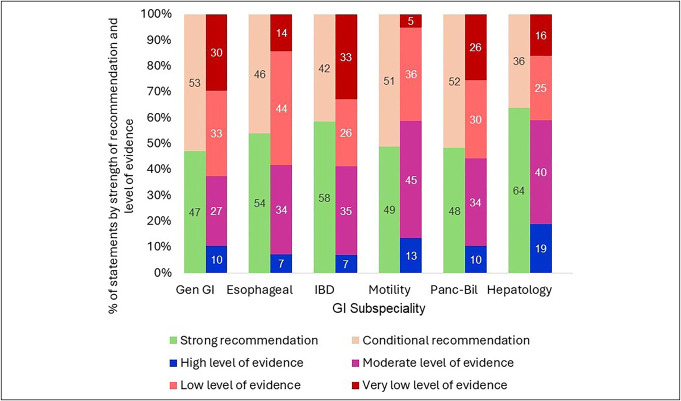
Strength of recommendation and level of evidence behind statements assessed by GRADE methodology by gastrointestinal subspecialty. GI, gastroenterology; GRADE, Grading of Recommendations, Assessment, Development, and Evaluation.

Of the GRADE guideline statements, 13% (327/2,609) had high, 37% moderate, 33% low, and 24% very low level of evidence supporting the statement. Most statements (52%) were strong recommendations, and 48% were conditional recommendations. Among the subspecialties, hepatology had the greatest proportion of statements that were strong recommendations (64%), followed by IBD (58%), esophageal (54%), motility (49%), pancreaticobiliary (48%), and general GI (47%) (Figure 2).

**Figure 3.**
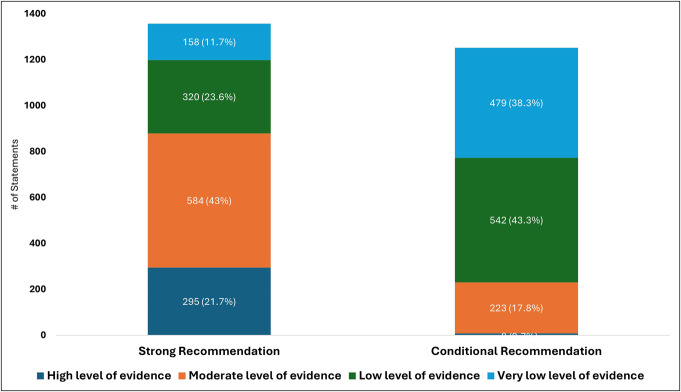
Level of evidence by strength of recommendation behind statements assessed by GRADE methodology. GRADE, Grading of Recommendations, Assessment, Development, and Evaluation.

The percentage of statements supported by the highest level of evidence was also highest within hepatology (19%), followed by 13% in motility, 10% in pancreaticobiliary and general GI, and the lowest (7%) in esophageal and IBD. Of the 82 statements assessed using the AHA/ACC methodology, 11% were level A, 28% at level B, with the majority (61%) classified as level C. Over time, there was a decline in the proportion of statements supported by the highest level of evidence across all specialties (Figures 1 and 3).

Of the statements with strong recommendations, 21% were supported by a high level of evidence, 43% by a moderate, 24% by low level, and 12% by very low levels of evidence. Of the statements with conditional recommendations, 0.7% were supported by a high, 18% by moderate, 43% by low, and 38% by very low levels of evidence.

## DISCUSSION

Although 52% of guideline statements in GI and hepatology were strongly recommended, only 13% of the recommendations were supported by high-level evidence. Hepatology had the highest proportion of statements with the highest level of evidence. Despite the increasing number of guideline statements published, the level of evidence supporting recommendations is declining over time.

In the era of evidence-based medicine, clinical practice guidelines are often seen as the fundamental tool for high-quality patient care (5). Our data reveal frequent discrepancies between the strength of the recommendation and level of evidence supporting the recommendation. This may be due to a particular clinical context and magnitude of risks and benefits (6). However, ensuring that guidelines are based on high levels of evidence remains imperative. If the trend seen in this study continues, guidelines may become a limited tool to guide evidence-based practices. This trend is not unique to GI. A 2020 study of cardiology guideline statements concluded that fewer than 10% of cardiology society guideline recommendations were supported by high-quality evidence (7).

Although the intention of these documents is to standardize care and practice, there is also an alarming trend of payors using guidelines to make decisions regarding reimbursement. A 2022 report by the Office of the Inspector General cited clinical guidelines as one reason for improper prior authorization denials by Medicare Advantage plans (8). This becomes a particular concern as there is often a lag between guideline development and clinical practice (9). Thus, future efforts need to determine how guideline statements can guide cost-effective care while balancing the need for individualized treatment decisions. Furthermore, updating guideline statements to reflect rapidly evolving medical knowledge will be vital.

Currently, the highest-level of evidence is RCTs, which are very expensive to conduct. It is impractical to generate RCT level of evidence for each recommendation. Generating novel clinical trial methodology to decrease the cost and increase the ability to study subgroups and secondary outcomes may result in lower-cost, higher-yield clinical trials. This may also allow for more investigator-initiated trials.

As with all retrospective analyses, there are several limitations to this study, including the inherent subjectivity of the GRADE methodology. There are risks of bias, inconsistency, and publication bias, which can affect the level of evidence (2). It is also subject to interpretation by the members reviewing these guidelines. A 2013 analysis of GI guidelines revealed that only 31% graded levels of evidence; of those that did 29% were supported by the highest quality of evidence (10). Although we have made progress in formally grading evidence, the proportion of statements supported by the highest quality of evidence has dropped. This analysis supports the need for GI societies to advocate for high-quality research to support the development of stronger evidence-based guideline statements.

## CONFLICTS OF INTEREST

**Guarantor of the article:** Bharati Kochar, MD, MS.

**Specific author contributions:** M.S.: generation, collection, analysis, and interpretation of data; drafting the manuscript. A.Y.: generation, collection, analysis, and interpretation of data; critical revision of the manuscript. N.N.U.: conception of the study, critical revision of the manuscript. A.C.: study design, critical revision of the manuscript. B.K.: study conception and design, drafting the manuscript and study supervision. Study data are publicly available.

**Financial support:** B.K. was supported by R03AG074059 and K76AG083309.

**Potential competing interests:** M.S., A.Y., N.N.U.: none. A.C.: consulting fees from Boehringer Ingelheim and Pfizer Inc. and grant support from Pfizer Inc. and Freenome. B.K.: advisory boards for Pfizer, Inc & Bristol Meyers Squibb.
